# Writing far and beyond the Thesis by the medical community

**DOI:** 10.12669/pjms.39.1.7197

**Published:** 2023

**Authors:** Anam Zafar, Afifa Ehsan

**Affiliations:** 1 Anam Zafar, Dr. Anam Zafar, BSPT, tDPT. Assistant Professor, Boston Physiotherapy and Wellness Clinic, Jail Road, Lahore, Pakistan; 2 Afifa Ehsan, Dr. Afifa Ehsan, BDS, M.Phil, CMT, CME, MHPE (Scholar). Department of Oral Biology, Faryal Dental College, Lahore, Pakistan

In doctoral academics, research writing and publishing is considered imperative criterion to appraise the performance of medical professionals, medical students, and medical institutions. Research publications are contemplated as the ladder to achieving academic promotions, acquiring funds for research purposes, and accomplishing better rankings of institutions. When research writings do not meet adequate quality to be published, it becomes the reason for contracting opportunities for professional exchange of ideas and professional growth, affecting not only individual perspectives but also on a larger scale limiting the enhancement of existing knowledge in the field. Nevertheless, it has been reported that medical publications contribute about 28% of the total output of research from Pakistan, from which 84% of the total research consists of original articles and Journal of Pakistan Medical Association (JPMA) and Pakistan Journal of Medical Sciences (PJMS) are at the top of the list of the trusted sources.^1^

Pakistan made promising progress in the number of publications in the last twenty years from 731 documents in 2001 to 6,685 documents in 2020. This inconsequential contribution to literature is of importance but publication outputs, in general, are quite low. A review of literature cited several reasons for low writing output from medical professionals which are a consequence of absence of literature reading practices, time limitation, dearth of motivation and momentum, lack of confidence and the absence of framework, recognized structures to endure, and habit of up keeping the writings.^2^

The notion behind compulsive thesis writing among medical professionals and students was predominantly to make them well versed with research culture and writing skills which are ultimately going to facilitate them in communicating with the outer world, with national and international colleagues, and sharing new ideas and knowledge with them. But this forced writing makes substandard documents on behalf of compulsive students and supervisors. In Pakistan, compulsive research documents are produced by persons who want professional excellence in their career compared to their colleagues and those individuals are mostly practitioners, not full-time researchers. Consequently, their writings have many inherent flaws such as biased population, prejudiced hypothesis formulation, and weaknesses in data collection.^3^

The whole scenario ultimately leads to a deficiency of motivation for doing authentic research among medical professionals and for their professional upgradation they are willing to get assistance by hiring professionals. These professionals are content writers or the purported thesis writers in the medical field preceding “ghostwriting” issues of authors which is detrimental to the field as it restricts the conveyance of valuable information to the audience. On the other hand, full-time researchers have proper reasoning for doing research and have a supportive team for the completion of their research process in a systematic manner minimizing all the biases and flaws coming in the way of original data which is the actual representative of the population of interest.^4^

Keeping this in view, the usage of arduous research approaches and their comprehension is indispensable for all medical practitioners, not only for a well-defined clinical decision-making process but also for the translation of theoretical knowledge into clinical practice, as well as communication between institutions, patients, and colleagues. It is of utmost importance to enhance the research culture in the medical community to contribute to the literature by emphasizing clinical judgment with inconsistencies between research findings and practice.

Research by choice is an endangered species of academic writing and a dilemma seen in our academics. Medical writing practices implore serious interventions from concerned authorities. This includes providing all the necessary digital support to universities and institutions i.e., subscription to different journals, launching of digital libraries, and high-speed internet facilities with new medical software and gadgets to improve access to updated medical literature. Designing new curriculum strategies to encourage research writing beyond the thesis may include early understanding of research rewarded with acknowledgment, and teaching research methodologies in early years with elective research activities. This may create more constructive approaches toward research writing and in the long run, more inherited research skills among practitioners. These strategies will facilitate students who can be good researchers not by compulsion but by their own choice and curiosity to contribute something valuable to literature.^5^

Challenging students intellectually will upsurge their self-determination and eventually these practices will make the effect so internalized that research writings will become naturally fascinating and gratifying for students and they will take up research by choice when they become practitioners. In the model of implementation of self-determination and curricular strategies, autonomy plays a vital role to increase the sense of competency, where competence alone is not adequate to warrant adherence among students for research curiosity, but rather, accompanies the increased autonomy. Autonomy can be increased by proposing students choose topics, supervisors, and group members by their own choices and effectively navigating their research process under mentorship. This suggests that curriculum strategies may lead to more operative learning and encourage future career involvement in research rather than just writing compulsively for dissertation and thesis.^6^

## Authors’ Contribution:

**AZ** wrote the first draft of the article which was subsequently revised by **AE**. Both authors approved the final submission.

## Note:

The opinion expressed in the present expert commentary is the view of the authors and does not necessarily reflect the view of the institutions.
